# In Vivo Imaging of Transplanted Islets Labeled with a Novel Cationic Nanoparticle

**DOI:** 10.1371/journal.pone.0057046

**Published:** 2013-02-22

**Authors:** Koichi Oishi, Yoshitaka Miyamoto, Hiroaki Saito, Katsutoshi Murase, Kenji Ono, Makoto Sawada, Masami Watanabe, Yasufumi Noguchi, Toshiyoshi Fujiwara, Shuji Hayashi, Hirofumi Noguchi

**Affiliations:** 1 Department of Advanced Medicine in Biotechnology and Robotics, Nagoya University Graduate School of Medicine, Nagoya, Aichi, Japan; 2 Nagoya Research Laboratory, MEITO Sangyo Co., Ltd., Nagoya, Aichi, Japan; 3 Department of Brain Functions, Division of Stress Adaptation and Protection, Research Institute of Environmental Medicine, Nagoya University, Nagoya, Aichi, Japan; 4 Center for Gene and Cell Therapy, Okayama University Graduate School of Medicine, Dentistry and Pharmaceutical Sciences, Okayama, Okayama, Japan; 5 Department of Socio-environmental Design, Hiroshima International University, Kure, Hiroshima, Japan; 6 Department of Gastroenterological Surgery, Okayama University Graduate School of Medicine, Dentistry and Pharmaceutical Sciences, Okayama, Okayama, Japan; Argonne National Laboratory, United States of America

## Abstract

To monitor pancreatic islet transplantation efficiency, reliable noninvasive imaging methods, such as magnetic resonance imaging (MRI) are needed. Although an efficient uptake of MRI contrast agent is required for islet cell labeling, commercially-available magnetic nanoparticles are not efficiently transduced into cells. We herein report the *in vivo* detection of transplanted islets labeled with a novel cationic nanoparticle that allowed for noninvasive monitoring of islet grafts in diabetic mice in real time. The positively-charged nanoparticles were transduced into a β-cell line, MIN6 cells, and into isolated islets for 1 hr. MRI showed a marked decrease in the signal intensity on T1- and T2-weighted images at the implantation site of the labeled MIN 6 cells or islets in the left kidneys of mice. These data suggest that the novel positively-charged nanoparticle could be useful to detect and monitor islet engraftment, which would greatly aid in the clinical management of islet transplant patients.

## Introduction

Pancreatic islet transplantation has become an option for the treatment of unstable type 1 diabetes [1]–[3]. The assessment of graft function is currently dependent on clinical biochemistry measurements, including the measurement of C-peptide levels, glucose levels, and oral/intravenous glucose tolerance tests [4]. Therefore, the establishment of a noninvasive technique for quantifying islet graft survival is extremely important for clinical islet transplantation. A promising approach might be positron emission tomography using ^18^F-fluorodeoxyglucose-labeled islets, especially in combination with computed tomography [5]. However, this method is limited by the short isotope half-life and low spatial resolution. Magnetic resonance imaging (MRI) is an attractive potential tool for measuring the islet mass *in vivo*, because it is generally noninvasive, it can achieve relatively high spatial resolution, and it can use multiple mechanisms for contrast enhancement [6]–[8]. However, the electromagnetic properties of the islet and liver tissue do not differ considerably, and therefore, labeling with an MR contrast agent is necessary for their discrimination [9].

Recently, the labeling of islet cells has been pursued with magnetic iron oxide particles and has allowed the detection of transplanted islets [10]–[16]. Such a technique could allow for real-time, noninvasive imaging of the post-transplanted viable islet mass and may facilitate the examination of various interventions to promote or sustain the islet mass over time. However, the commercially-available magnetic nanoparticles are not efficiently transduced into cells because of their negative charge, since the cell surface is normally negatively charged. We recently developed six kinds of novel magnetic iron oxide nanoparticles, which are electrically-charged by a cationic end-group substitution of dextran [17], [18]. Each of the nanoparticles consists of a small monocrystalline, superparamagnetic iron oxide core that is stabilized by a cross-linked aminated dextran coating to improve its stability. Cultured cells were efficiently labeled with one of the positively-charged nanoparticles, TMADM-03, but not with commercially-available nanoparticles [18]. These data suggest that the cationic nanoparticle could be useful as a MRI contrast agent to monitor the islet mass after transplantation.

In this study, we report on the *in vivo* detection of transplanted islets labeled with a cationic nanoparticle that allowed for noninvasive monitoring of islet grafts in diabetic mice in real time.

## Materials and Methods

### Animals

Eight-week-old male athymic BALB/c nude mice weighing 25–30 g, and six-week-old male adult Sprague-Dawley (SD) rats weighing 250–300 g were purchased from SLC Japan. The mice and rats were housed under specific pathogen-free conditions with a 12 h light/dark cycle and had free access to food and water. The mouse and rat studies were approved by the review committee of Nagoya University Graduate School of Medicine and Okayama University Graduate School of Medicine, Dentistry and Pharmaceutical Sciences.

### Cell line

MIN6 cells, kindly provided by Dr. Hideaki Kaneto (Department of Internal Medicine, Osaka University, Japan [19]), were routinely grown in sterile plastic flasks containing Dulbecco's modified Eagle's medium (DMEM) and 25 mM glucose supplemented with 15% fetal bovine serum (FBS), 100 U/ml penicillin and 100 µg/ml streptomycin and 5 µ/L β-mercaptoethanol at 37°C in a humidified atmosphere of 5% CO_2_.

### Cell labeling and estimation of the iron content in MIN6 cells

Trimethylamino dextran-coated, magnetic iron oxide nanoparticles (TMADM-03) were kindly provided by MEITO Sangyo Co., Ltd. (Kiyosu, Japan). MIN6 cells were detached from the plates using Trypsin-EDTA and incubated for several hours, at several temperatures, with each nanoparticle reconstituted in DMEM with or without 15% FBS. At the end of the uptake experiments, the cells were washed 3 times in phosphate-buffered saline (PBS). Measurement of cellular toxicity was performed by the manual counting method based on the trypan blue exclusion procedure. The iron content of MIN6 cells labeled with each nanoparticle was measured by photon correlation spectroscopy (PCS), using a Nuclear Magnetic Resonance (NMR) Sequence (Autosizer 4700: Malvern Instruments, Orsay, France) at 90° with the Contin measurement method [20]. At the end of the uptake experiment, labeled cells were collected in 500 µL deionized water and homogenized. The volume was brought up to 1 mL with deionized water and analyzed by pulse NMR.

### Electron microscopy

Electron microscopy was used to visualize the presence of iron-oxide nanoparticles inside the MIN6 cells. MIN6 cells labeled with TMADM-03 were fixed with 2% paraformaldehyde and 2% glutaraldehyde in 0.1 M phosphate buffer (pH 7.4) at 4°C for 24 hr, followed by incubation in 2% osmium tetroxide at 4°C for 90 min. The cells were dehydrated in increasing concentrations of ethanol, immersed in propylenoxide, and embedded in Quetol 812 (Nissin EM, Tokyo). Ultrathin sections (70 nm) were stained using Reynold's lead citrate and examined using a JEM-1200EX transmission electron microscope (JOEL, Ltd., Tokyo) at an accelerating voltage of 80 kV.

### Islet isolation, labeling, and transplantation

Islet isolation was performed as follows: under general anesthesia induced by pentobarbital sodium (50 mg/kg), rats were injected with 10 mL of Hanks' balanced salt solution (Gibco) containing 2 mg/mL collagenase (Sigma; type V) into the common bile duct. The distended pancreas was removed and incubated at 37°C for 16 min. The islets were purified by centrifugation (2000 rpm for 10 min) with Histopaque 1077- RPMI 1640 medium gradient (Sigma). Individual islets, free of attached acinar, vascular, and ductal tissues, were selected and removed with a Pasteur pipette under a dissecting microscope, yielding highly purified islets for transplantation. The crude number of islets in each diameter class was determined by counting the islets using an optical graticule. The crude number of islets was then converted to the standard number of islet equivalents (IE; diameter standardizing to 150 µm) [21]. The islets were incubated for 1 hr at 37°C with TMADM-03 reconstituted in DMEM with 15% FBS. At the end of the uptake experiments, islets were washed 3 times in RPMI 1640 medium. The islets were transplanted into the renal subcapsular space of the left kidney of a diabetic nude mouse. Transplantation of MIN6 cells labeled with TMADM-03 was performed by the same method as that used for islet transplantation.

### MRI

Mice were lightly anesthetized using isoflurane (3% induction and 1.5% maintenance) and held in place with an MRI coil. The coil was set on MRI equipment (MRTechnology, Inc., Tsukuba, Japan) and T1 and T2-weighted images were acquired according to the manufacture's protocol. All T1- and T2-weighted image data sets were visually evaluated to identify the location of the transplanted cells within each animal.

### In vitro evaluation of isolated islets

The islet viability was assessed using double fluorescein diacetate/propidium iodide (FDA/PI) staining to visualize living and dead islet cells simultaneously [1], [2], [21]. Fifty islets were inspected and their individual viability was determined visually, followed by calculation of their average viability. Islet function was assessed by monitoring the insulin secretory response of the purified islets during glucose stimulation using a procedure described by Shapiro and colleagues [1], [2]. Briefly, 1200 IE were incubated with either 2.8 mM or 25 mM glucose in RPMI 1640 for 2 hr at 37°C and 5% CO_2_. The supernatants were collected, and insulin levels were determined using a commercially available enzyme-linked immunosorbent assay (ELISA) kit (ALPCO Insulin ELISA kit; ALPCO Diagnostics, Windham, NH). The stimulation index was calculated by determining the ratio of insulin released from islets incubated in a high glucose concentration to the insulin released by the islets in the low glucose concentration. The data were normalized to the total DNA. The data were expressed as the means ± SE.

### In vivo assessment

Islets labeled with or without TMADM-03 were transplanted into the renal subcapsular space of the left kidney of a diabetic nude mouse, as described above. During the 30-day post-transplantation period, the non-fasting blood glucose levels were monitored 3 times per week. Normoglycemia was defined when 2 consecutive blood glucose level measurements were less than 200 mg/dl. The intra-peritoneal glucose tolerance test (IPGTT) was performed thirty days after transplantation. The mice were fasted overnight, after which glucose (2.0 g/kg body weight) was injected intraperitoneally. The blood glucose levels were measured before injection and at 5, 30, 60, 90, and 120 minutes after injection.

### Statistics

The differences between each group were considered significant if the value was p<0.01 using an unpaired Student's *t*-test with Bonferroni correction or the Kaplan–Meier log-rank test.

## Results

### Conditions of cell labeling

**Figure 1:**
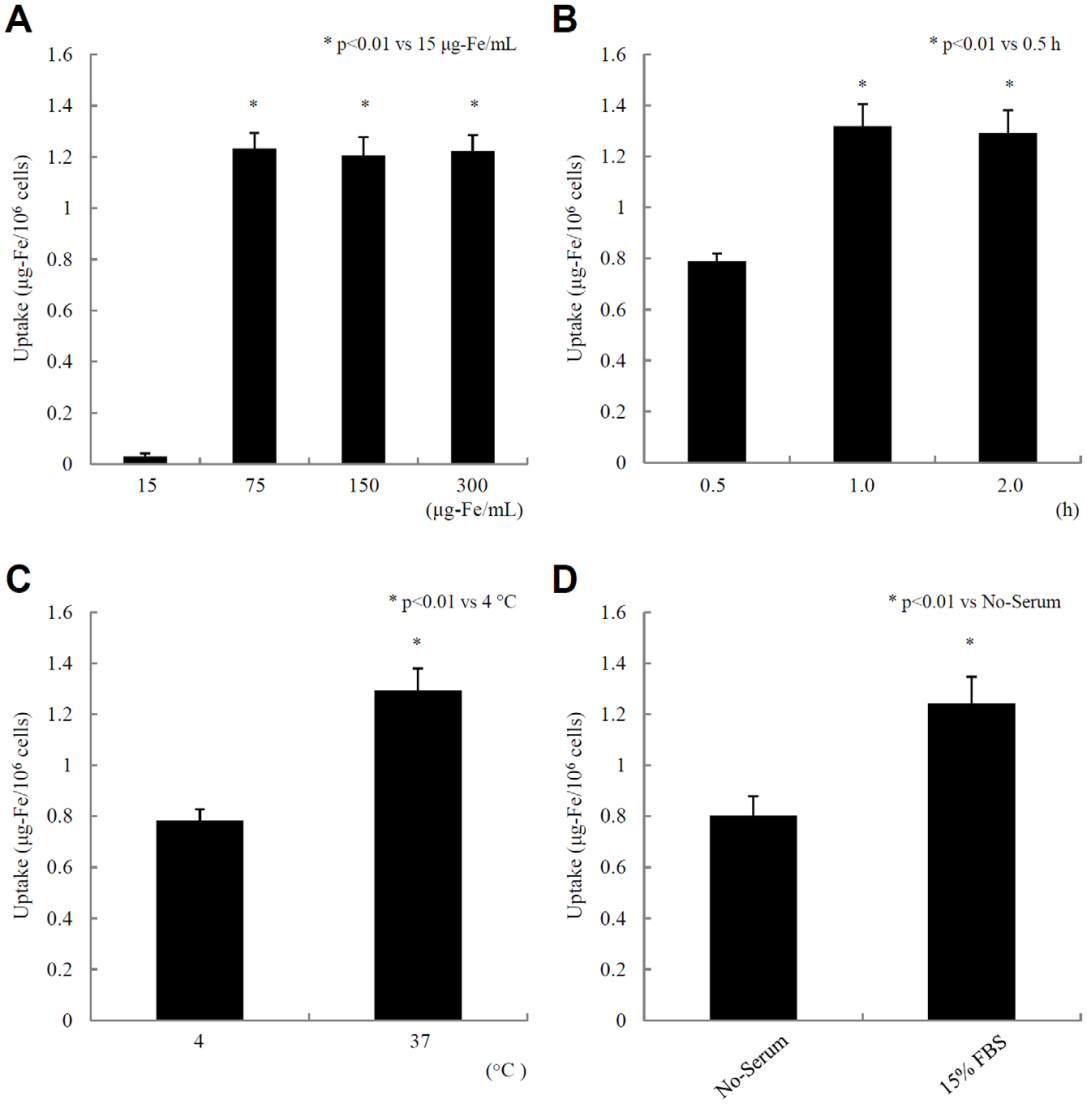
The conditions for cell labeling with TMADM-03. **A.** Concentration studies. MIN6 cells were incubated for 1 hr at 37°C with several concentrations of the contrast agent. **B.** Time course studies. MIN6 cells were incubated at 37°C with 150 µg-Fe/mL TMADM-03 for different lengths of time. **C.** Temperature studies. MIN6 cells were incubated at 4°C or 37°C for 1 hr with 150 µg-Fe/mL TMADM-03. **D.** Medium studies. MIN6 cells were incubated at 37°C for 1 hr with 150 µg-Fe/mL TMADM-03 in culture medium with or without 15% FBS.

We recently developed six kinds of novel cationic nanoparticles [17], [18] and found that cells were efficiently labeled with one of the positively-charged nanoparticles, TMADM-03, *in vitro* [18]. Therefore, we used TMADM-03 in this study (Table 1). To determine the quality of cell labeling with TMADM-03, MIN6 cells were incubated for 1 hr at 37°C with several concentrations of the contrast agent. The uptake of the nanoparticle reached its peak in the 75 µg-Fe/mL reaction (Fig. 1A). Next, the cells were incubated at 37°C with 150 µg-Fe/mL of TMADM-03 for several hrs. The iron uptake reached its peak by 1 hr (Fig. 1B). To examine the temperature-dependence of the uptake, the cells were incubated at 4°C or 37°C for 1 hr with 150 µg-Fe/mL of TMADM-03. There was a more efficient uptake at 37°C than at 4°C (Fig. 1C). Finally, the cells were incubated at 37°C for 1 hr with 150 µg-Fe/mL of TMADM-03 in culture medium with or without 15% FBS. There was a more efficient uptake with 15% FBS than without FBS (Fig. 1D). These data suggest that the best condition for cell labeling by TMADM-03 is a 1 hr incubation at 37°C with more than 75 µg-Fe/mL of nanoparticles in the culture medium containing 15% FBS.

**Table 1:** Characterization of magnetic nanoparticles.

Sample name	coated dextran analog		magnetic nanoparticle				uptake
	substitution terminal	substitution rate	Dx/Fe	Diameter (nm)	R_2_ (mM·sec)^−1^	Zeta Voltage (mV)	(mg-Fe/10^6^cell)
ATDM	-	0	0.3	68	175	−15	0.2
TMADM-03	-N(CH3)_3_	0.24	1.4	44	148	+2	1.5

ATDM: a commercially available contrast agent (Resovist®).

### Electron microscopy

**Figure 2:**
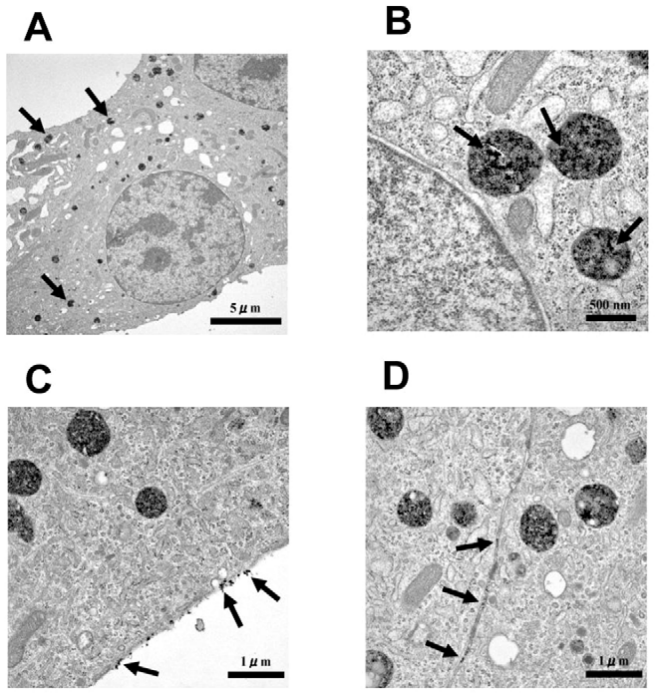
Electron microscopy of MIN6 cells labeled with TMADM-03. **A.** The morphology of the MIN6 cells labeled with TMADM-03. The arrows point to lysosomes containing the nanoparticles; ×4,860. **B.** Nanoparticles (arrows) detected in the lysosomes; ×30,200. **C.** Nanoparticles (arrows) attached to the cell membrane; ×18,400. **D.** Nanoparticles (arrows) trapped between cells; ×18,400.

Transmission electron microscopy (TEM) confirmed the presence of iron-oxide nanoparticles inside the MIN6 cells. Nanoparticles were found in different cell structures and were mainly observed in lysosomes (Figs. 2A, B). Of particular interest was the observation that the nanoparticles were either attached to the cell membrane (Fig. 2C) or trapped between the cells (Fig. 2D).

### In vivo MR imaging of transplanted MIN6 cells labeled with TMADM-03

**Figure 3:**
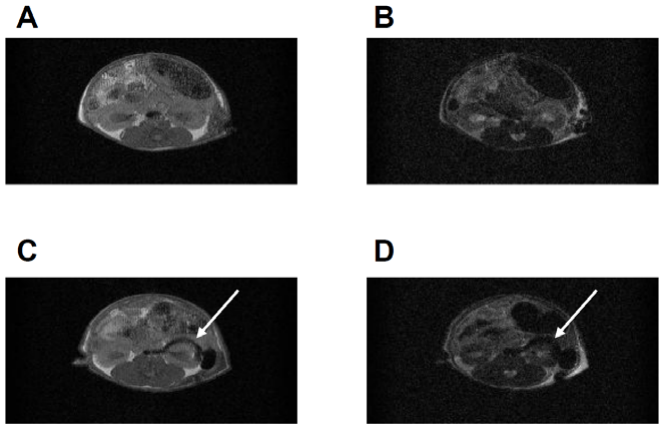
In vivo MRI of transplanted MIN6 cells labeled with TMADM-03. **A.** T1-weighted images of MIN6 cells without TMADM-03 transplanted into the left kidney capsule. **B.** T2-weighted images of MIN6 cells without TMADM-03 transplanted into the left kidney capsule. **C.** T1-weighted images of MIN6 cells labeled with TMADM-03 transplanted into the left kidney capsule. **D.** T2-weighted images of MIN6 cells labeled with TMADM-03 transplanted into the left kidney capsule. The arrows show the transplanted graft.

To assess the feasibility of *in vivo* imaging of transplanted cells in mice, 5×10^6^ MIN6 cells were incubated for 1 hr at 37°C in culture medium containing 15% FBS with or without 150 µg-Fe/mL TMADM-03. The cells were then transplanted under the left kidney capsule of diabetic mice. MRI showed a marked decrease in signal intensity on T1- and T2-weighted images at the implantation site of mice transplanted with labeled MIN6 cells (Figs. 3A–D).

### In vivo MR imaging of transplanted islets labeled with TMADM-03

**Figure 4:**
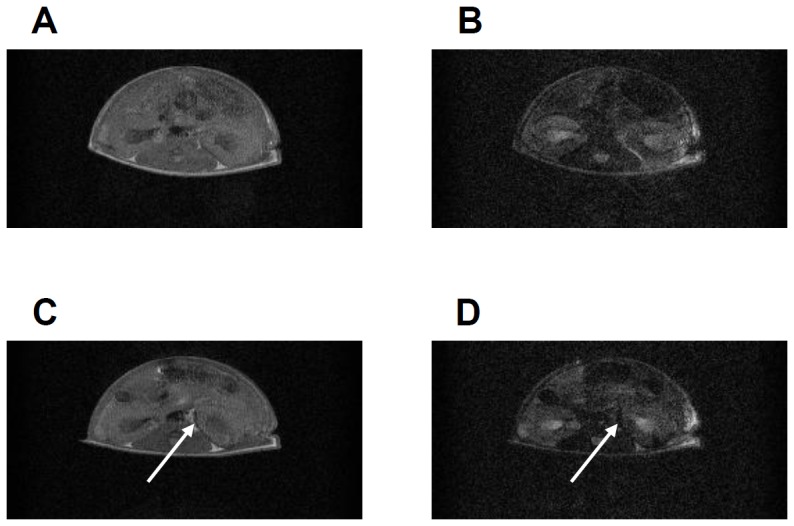
In vivo MRI of transplanted islets labeled with TMADM-03. **A.** T1-weighted images of unlabeled islets (1000 IEs) transplanted into the left kidney capsule. **B.** T2-weighted images of unlabeled islets (1000 IEs) transplanted into the left kidney capsule. **C.** T1-weighted images of islets (1000 IEs) that were labeled with TMADM-03 and transplanted into the left kidney capsule. **D.** T2-weighted images of islets (1000 IEs) that were labeled with TMADM-03 and transplanted into the left kidney capsule. The arrows show transplanted grafts.

Based on the data using MIN6 cells, we assessed the feasibility of the *in vivo* imaging of transplanted islets in mice. A total of 1000 IE of isolated islets were incubated for 1 hr at 37°C in culture medium containing 15% FBS with or without 150 µg-Fe/mL of TMADM-03 and transplanted under the left kidney capsule of diabetic mice. MRI again showed a marked decrease in the signal intensity on T1- and T2-weighted images at the implantation site of mice transplanted with labeled islets (Figs. 4A–D). These data suggest that TMADM-03, a positively-charged nanoparticle, could be useful for *in vivo* imaging.

### In vitro assessment of isolated islets labeled with TMADM-03

**Figure 5:**
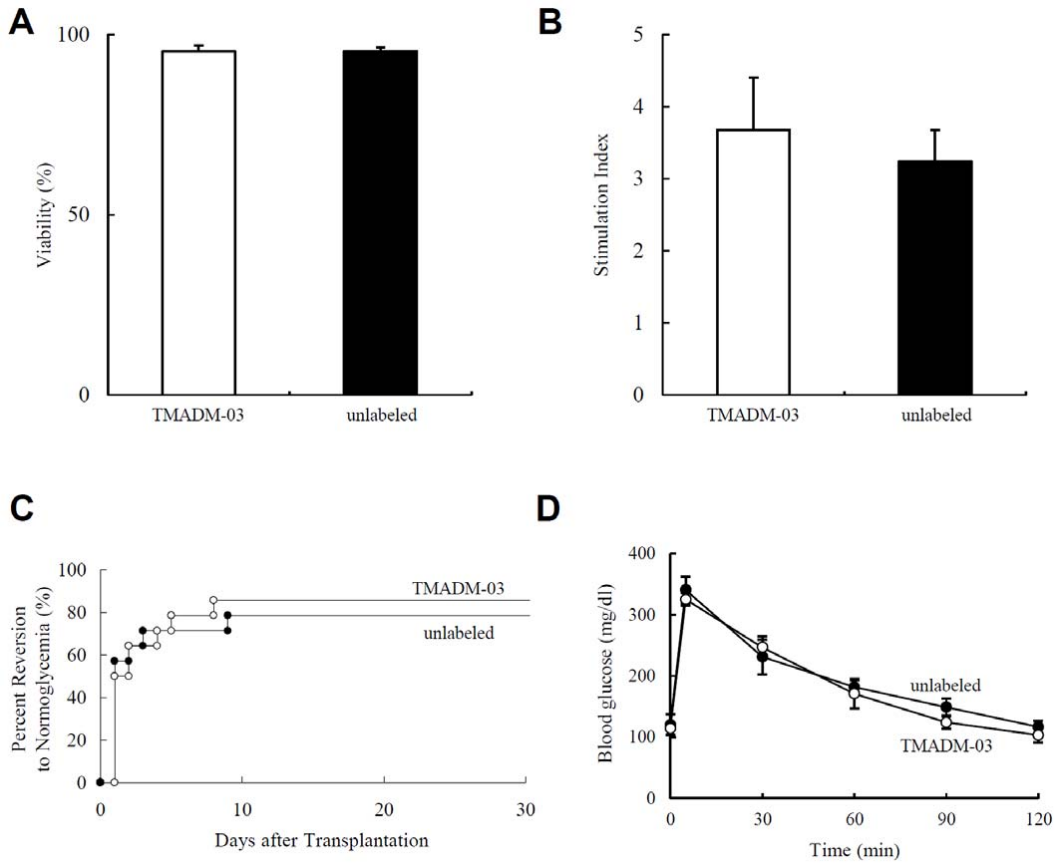
In vitro and in vivo evaluation of isolated islets labeled with TMADM-03. **A.** Islet viability. The viability of islets with or without the TMADM-03 label was assessed using FDA/PI staining. Fifty islets were inspected, and their individual viability was determined visually, followed by calculation of their average viability. **B.** Stimulation index. The islet potency was assessed by a static glucose challenge. TMADM-03-labeled islets, n = 5; Unlabeled islets, n = 5. **C.** The percentage of STZ-induced diabetic nude mice achieving normoglycemia after islet transplantation. A total of 1000 IE were transplanted below the kidney capsule of diabetic nude mice. Normoglycemia was defined as two consecutive post-transplant blood glucose levels of less than 200 mg/dl. TMADM-03-labeled islets, n = 14; Unlabeled islets, n = 14. **D.** IPGTTs in mice from each group. The IPGTT was performed thirty days after transplantation. The mice were fasted overnight, then glucose (2.0 g/kg body weight) was injected intraperitoneally. The blood glucose levels were measured before injection and at 5, 30, 60, 90, and 120 minutes after injection. No statistically significant differences in either the pre-transplantation blood glucose levels or pre-transplantation body weight were observed between the two groups of mice. TMADM-03-labeled islets, n = 4; Unlabeled islets, n = 4.

To assess the viability of islets labeled with or without TMADM-03 *in vitro*, the FDA/PI staining of isolated islets was measured. The islet viability evaluated by FDA/PI staining showed no significant differences between islets labeled with TMADM-03 (95.3±1.6%) and unlabeled islets (95.3±1.2%; Fig. 5A). The islet potency was assessed by a static glucose challenge *in vitro*. There were no significant differences in the stimulation index between the two groups (TMADM-03-labeled islets, 3.7±0.7; unlabeled islets, 3.2±0.4; Fig. 5B). These data suggest that the islet viability and function are not altered by the additional labeling step.

### In vivo assessment of isolated islets labeled with TMADM-03

To assess the graft function of islets labeled with or without TMADM-03 *in vivo*, 1000 IE from each group were transplanted below the kidney capsule of STZ-induced diabetic nude mice. No statistically significant differences in either the pre-transplantation blood glucose levels or the pre-transplantation body weight were observed between the two groups of mice. The blood glucose levels of 12 of the 14 mice (85.7%) that received islets labeled with TMADM-03 and 11 of the 14 mice (78.5%) that received islets without TMADM-03 decreased gradually and eventually reached normoglycemia (Fig. 5C). The blood glucose levels remained stable thereafter and returned to the pre-transplantation levels after the islet-bearing kidneys were removed (30 days after transplantation). The attainability of post-transplantation normoglycemia was similar between the two groups. The IPGTT was carried out on both groups thirty days after transplantation. The mice in both groups that were hyperglycemic were excluded. The mice were fasted overnight, after which glucose (2.0 g/kg body weight) was injected intraperitoneally. There were no significant differences in the blood glucose levels of mice after injection between the two groups (Fig. 5D). These data suggest that islet labeling with TMADM-03 does not affect their function *in vitro* or *in vivo*.

### Labeling efficiency by TMADM-03 compared with other compounds

**Figure 6:**
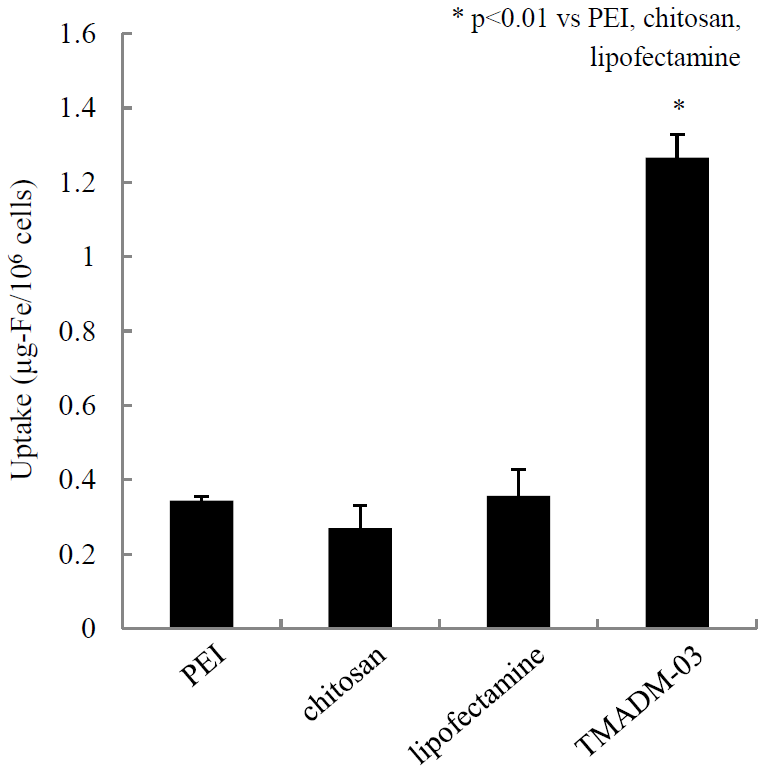
The labeling efficiency of TMADM-03 compared with other compounds. TMADM-03 and nanoparticles with PEI, chitosan, and cationic lipid (lipofectamine) were evaluated for their labeling efficiency. The conditions used for cell labeling was a 1 hr incubation at 37°C in culture medium containing 15% FBS. The concentration of TMADM-03 was 150 µg-Fe/mL, and the concentrations of the nanoparticles with other compounds were the same as in the previous reports [21]–[23].

Recently, it was reported that polyethylene imine (PEI) [22], chitosan [23], and cationic lipid [24] were coated on the surface of superparamagnetic iron oxide nanoparticles for the same purpose. Therefore, we compared the labeling efficiency provided by these compounds to that of TMADM-03. The condition used for cell labeling was a 1 hr incubation at 37°C in the culture medium containing 15% FBS. The concentration of TMADM-03 was 150 µg-Fe/mL, and the concentrations of the nanoparticles with other compounds were the same as in the previous reports [22]–[24]. With regard to the uptake of the nanoparticles, TMADM-03 provided the highest rate out of these compounds (Fig. 6).

## Discussion

For islet transplantation, islets from two or more donors are usually needed to achieve at least a transient insulin-independent state, and the long-term outcomes are still not satisfactory in terms of insulin independence. Potential causes of failure of islet transplants include failure of the initial engraftment, instant blood-mediated inflammatory reaction, allo- or auto-immune responses, glucotoxicity, and β-cell toxicity mediated by immunosuppressive agents [4], [25], [26]. In this study, we showed the positively charged nanoparticle, TMADM-03, to be efficiently transduced into cells and thus it was able to be used to visualize the transplanted cells. The time required to label the cells with TMADM-03 is only 1 hr, and the conditions for cell labeling are the same as the normal culture condition for islets (37°C, 5% CO_2_, normal culture medium). It was reported that islet visualization by a MR contrast agent, ferucarbotran was less efficient in humans when the labeling period was less than 16 hr [16], thus demonstrating the superiority of TMADM-03. Moreover, islet labeling with TMADM-03 does not affect their functions *in vitro* or *in vivo*. Therefore, TMADM-03 could be useful to detect and monitor islet engraftment, which would greatly aid in the clinical management of diabetes.

It has been reported that magnetic iron oxide nanoparticles covered with a modified dextran coating could be derivatized with a cell penetrating peptide [27]. Several such peptides have recently been described [19], [28]–[31]. Most of these peptides have positively charged amino acids, such as arginine and lysine. Therefore, we used TMADM-03, which is positively charged, for cell labeling. The best labeling results were achieved following incubation for 1 hr at 37°C in a serum-containing medium with the contrast agent. Moreover, TEM confirmed the presence of iron-oxide nanoparticles in cell lysosomes, as shown in Fig. 2. Additionally, the treatment of islets with amiloride, a specific inhibitor of the Na^+^/H^+^ exchange required for macropinocytosis, resulted in a reduction in the cell labeling (data not shown). These data suggest that cell labeling by the positively charged nanoparticles may depend on macropinocytosis, by which positively charged cell-penetrating peptides are transduced into cells.

It was previously reported that islets were efficiently labeled with PEI- [22], chitosan- [23], or cationic lipid- [24] coated nanoparticles. Therefore, we evaluated the labeling efficiency of TMADM-03 compared with these compounds. As shown in Fig. 6, TMADM-03 had the highest uptake of the nanoparticles out of these compounds.It was reported that islets were cultured with PEI-coated nanoparticle for 24 hrs [22], with chitosan-coated nanoparticles overnight [23], and with cationic lipid-coated nanoparticles for 24 hrs [24]. Therefore, the lower efficiency of the nanoparticles made with these compounds compared with TMADM-03 may be due to the insufficient incubation time used for the labeling. In other words, one of the advantages of TMADM-03 is short time needed for cell labeling.

In this study, we used mouse islets, while labeling of human islets was not performed. Moreover, islets were transplanted into the renal subcapsular space, which is a site that is not normally used in clinical islet transplantation. Intraportal placement in liver which is a site which is normally used in clinical islet transplantation, would lead to the occurrence of more artifacts due to the high iron content of the liver. We will add TMADM-03 to human islets using the intraportal transplant model in future studies. We will also investigate allorejection model to demonstrate how the signal is observed the extinguished in temporal association with rejection.

We conclude that TMADM-03, which is a modified form of a commercially available contrast agent, Resovist® (ATDM), can be used as a marker of isolated pancreatic islets for detection by MRI. Following transplantation into the kidneys of mice, the labeled pancreatic islets could be easily detected following transplantation as less intense regions on both T1- and T2-weighted MR images. This approach could potentially be translated into clinical practice for evaluating graft survival and for monitoring therapeutic intervention during graft rejection.
